# Gene model for the ortholog of *Sik3* in *Drosophila mojavensis*

**DOI:** 10.17912/micropub.biology.001032

**Published:** 2025-04-01

**Authors:** Gabriella N. Bicanovsky, Karolina J. Senkow, Cassidy McColl, Jennifer Mierisch, Kellie S. Agrimson, Lindsey J. Long, Judith Leatherman, Chinmay P. Rele, Laura K Reed

**Affiliations:** 1 The University of Alabama, Tuscaloosa, AL USA; 2 Loyola University Chicago, Chicago, IL USA; 3 Saint Catherine University, Saint Paul, MN, USA; 4 Oklahoma Christian University, Edmond, OK USA; 5 University of Northern Colorado, Greeley, CO USA

## Abstract

Gene model for the ortholog of *Salt-inducible kinase 3* ( *Sik3* ) in the May 2011 (Agencourt dmoj_caf1/DmojCAF1) Genome Assembly (GenBank Accession: GCA_000005175.1 ) of *Drosophila mojavensis* . This ortholog was characterized as part of a developing dataset to study the evolution of the Insulin/insulin-like growth factor signaling pathway (IIS) across the genus *Drosophila* using the Genomics Education Partnership gene annotation protocol for Course-based Undergraduate Research Experiences.

**Figure 1. Genomic neighborhood and gene model for Sik3 in Drosophila mojavensis:**
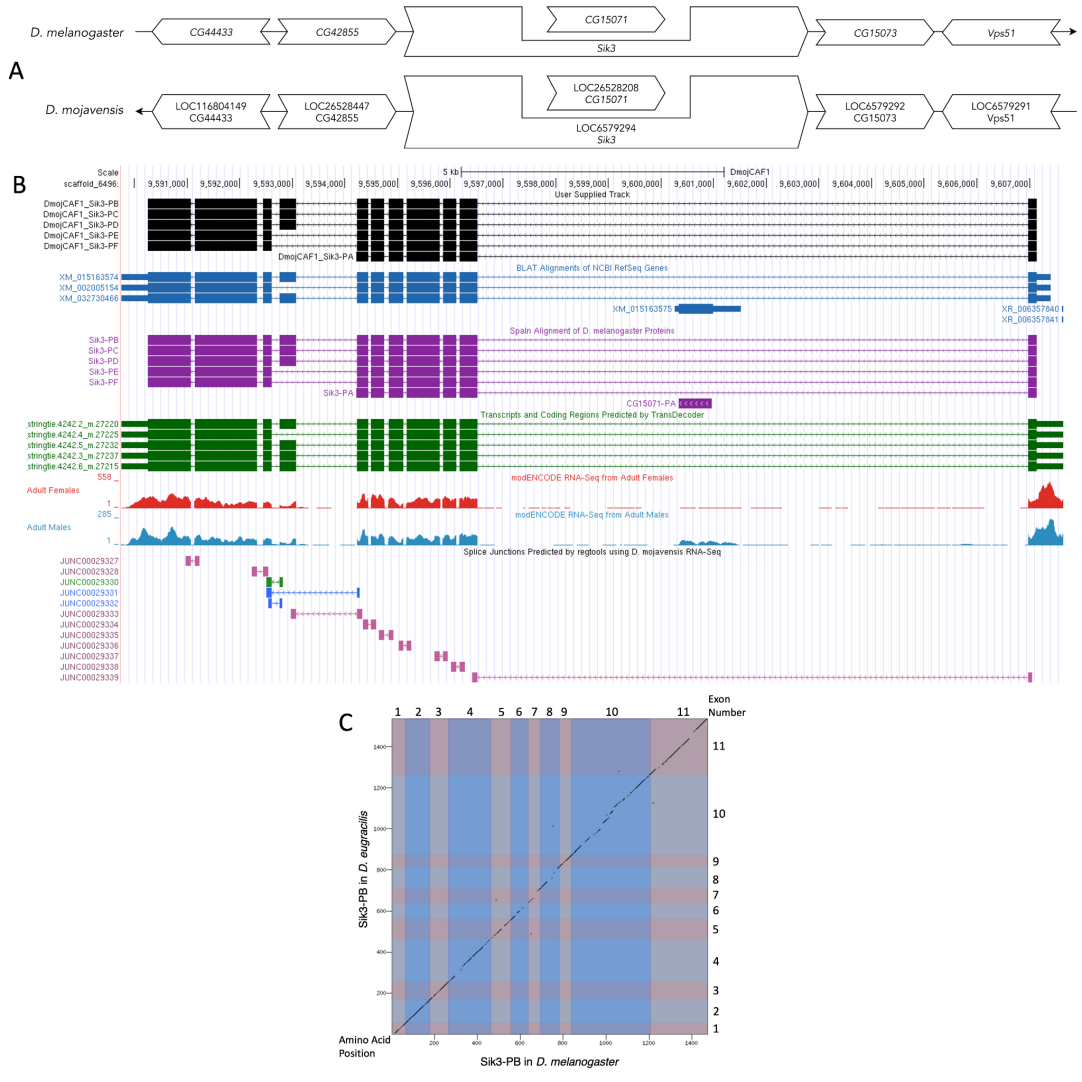
**
(A) Synteny of genomic neighborhood of *Sik3* in *D. melanogaster* and *D. mojavensis* .** Gene arrows pointing in the same direction as reference gene in both *D. mojavensis* and *D. melanogaster* are on the same strand as the target gene; gene arrows pointing in the opposite direction are on the opposite strand. The thin underlying arrow pointing to the right indicates that *Sik3* is on the + strand in *D. melanogaster* ; arrow pointing to the left indicates that *Sik3* is on the – strand in *D. mojavensis* . White arrows in *D. mojavensis* indicate the locus ID and the orthology to the corresponding gene in *D. melanogaster* . The gene names given in the *D. mojavensis* gene arrows indicate the orthologous gene in *D. melanogaster* , while the locus identifiers are specific to *D. mojavensis* . **(B) Gene Model in UCSC Track Hub (Raney et al., 2014):** the gene model in *D. mojavensis* (black), Spaln of *D. melanogaster* Proteins (purple, alignment of Ref-Seq proteins from *D. melanogaster* ), BLAT alignments of NCBI Ref-Seq Genes (blue, alignment of Ref-Seq genes for *D. mojavensis* ), RNA-Seq from Adult Females (red) and Adult Males (blue, alignment of Illumina RNA-Seq reads from *D. mojavensis* ), and Transcripts (green) including coding regions predicted by TransDecoder and Splice Junctions Predicted by regtools using *D. mojavensis* RNA-Seq (SRP006203) Splice junctions shown have a minimum read-depth of 11 with 10-49, 50-99, 100-499 supporting reads in blue, green, pink, respectively. The custom gene model (User Supplied Track) is indicated in black with CDS depicted with wide boxes, intron with narrow lines (arrows indicate direction of transcription). **(C) Dot Plot of Sik3-PB in *D. melanogaster* ( *x* -axis) vs. the orthologous peptide in *D. mojavensis* ( *y* -axis).** Amino acid numbers are indicated along the left and bottom; CDS numbers are indicated along the top and right, and CDSs are also highlighted with alternating colors.

## Description

**Table:** 


* This article reports a predicted gene model generated by undergraduate work using a structured gene model annotation protocol defined by the Genomics Education Partnership (GEP; thegep.org ) for Course-based Undergraduate Research Experience (CURE). The following information in this box may be repeated in other articles submitted by participants using the same GEP CURE protocol for annotating Drosophila species orthologs of Drosophila melanogaster genes in the insulin signaling pathway. * "In this GEP CURE protocol students use web-based tools to manually annotate genes in non-model *Drosophila* species based on orthology to genes in the well-annotated model organism fruitfly *Drosophila melanogaster* . The GEP uses web-based tools to allow undergraduates to participate in course-based research by generating manual annotations of genes in non-model species (Rele et al., 2023). Computational-based gene predictions in any organism are often improved by careful manual annotation and curation, allowing for more accurate analyses of gene and genome evolution (Mudge and Harrow 2016; Tello-Ruiz et al., 2019). These models of orthologous genes across species, such as the one presented here, then provide a reliable basis for further evolutionary genomic analyses when made available to the scientific community.” (Myers et al., 2024). “The particular gene ortholog described here was characterized as part of a developing dataset to study the evolution of the Insulin/insulin-like growth factor signaling pathway (IIS) across the genus *Drosophila* . The Insulin/insulin-like growth factor signaling pathway (IIS) is a highly conserved signaling pathway in animals and is central to mediating organismal responses to nutrients (Hietakangas and Cohen 2009; Grewal 2009).” (Myers et al., 2024). “ *D.* *mojavensis* (NCBI:txid7230) is part of the *mulleri complex* in the *repleta* species group within the subgenus *Drosophila* of the genus *Drosophila* (Wasserman 1992; Durando et al., 2000) *.* It was first described by Patterson (Patterson and Crow 1940). *D. mojavensis* specializes on rotting cactus as its host and is found in the Mojave and Sonoran Deserts of the southwestern United States and northwestern Mexico including the Baja Peninsula, as well as on the channel-islands off the coast of California (https://www.taxodros.uzh.ch, accessed 1 Feb 2023).” (Congleton et al., 2023).

We propose a gene model for the *D. mojavensis* ortholog of the *D. melanogaster* *Salt-inducible kinase 3* ( *Sik3* ) gene. The genomic region of the ortholog corresponds to the uncharacterized protein XP_015019060.1 (Locus ID LOC6579294 ) in the May 2011 (Agencourt dmoj_caf1/DmojCAF1) Genome Assembly of *D. mojavensis* ( GCA_000005175.1 ). This model is based on RNA-Seq data from *D. mojavensis* ( SRP006203 - Chen et al., 2014) and *Sik3* in *D. melanogaster* using FlyBase release FB2023_03 ( GCA_000001215.4 ; Larkin et al., 2021; Gramates et al., 2022; Jenkins et al., 2022).

The gene *Sik3* ( *Salt-inducible kinase 3* ) is related to the AMPK Ser/Thr class of kinases and was identified using sequence homology upon comparison of the human and mouse *SIK* genes with the *Drosophila melanogaster* genome (Okamoto et al., 2004). *Sik3* null mutants are not viable, so studies in *D. melanogaster* used hypomorphic alleles to characterize the role of Sik3 in the Insulin/TOR pathway (Wang et al., 2011). Under fed conditions, Sik3 is phosphorylated and activated downstream of Akt and liver kinase B1 (LKB1) where Sik3 promotes sequestration of Histone Deacetylase 4 (HDAC4) in the cytoplasm via phosphorylation (Wang et al., 2011; Choi et al., 2015). Under fasting conditions, Protein Kinase A (PKA) phosphorylates and inactivates Sik3, allowing HDAC4 to translocate to the nucleus and activate the transcription factor Forkhead Box, subgroup O (dFOXO) (Walkinshaw et al., 2013; Wang et al., 2011). Sik3 has also been shown to negatively regulate the Hippo signaling pathway in *Drosophila* and to be involved in circadian rhythm regulation in a range of species from flies to mice (Wehr et al., 2013; Funato et al., 2016; Liu et al., 2022).

***Synteny***

*Sik3* occurs on chromosome 2R in *D. melanogaster* and is flanked by upstream genes *CG44433 , CG42855 ,* and downstream genes *CG15073 , Vacuolar protein sorting 51* ( *Vps51* ) *. Sik3* holds a nested gene of *CG15071 .* It has been determined that the putative ortholog of *Sik3* is found on scaffold CH933808.1 (scaffold_6496) in *D. mojavensis* with LOC6579294 ( XP_015019060.1 , via *tblastn* search with an e-value of 0.0 and percent identity of 67.19%), where it is surrounded by upstream genes LOC116804149 ( XP_032586360.1 ) and LOC26528447 ( XP_015019062.1 ), which correspond to *CG44433* and *CG42855* in *D. melanogaster* with e-values 0.48, 7e-11 and and percent identities of 65.00%, 52.63%, respectively, as determined by *blastp* ( Figure 1A, Altschul et al., 1990). The nested gene within the putative ortholog has a LOCID of LOC26528208 ( XP_015019061.1 ) and it corresponds to *CG15071* in *D. melanogaster* with an e-value of 4e-56 and a percent identity of 52.15%. The putative ortholog is flanked downstream by LOC6579292 ( XP_002005188.1 ) and LOC6579291 ( XP_032586692.1 ), which correspond to *CG15073* and *Vps51* in *D. melanogaster* with e-values of 0.0, and percent identities 60.22%, 91.87%, respectively, as determined by *blastp* . This is likely the correct ortholog assignment for *Sik3* in *D. mojavensis* for two reasons: 1) the best alignment indicated with a *blastp* search resulting in *Sik3* with an e-value of 0.0 and a percent identity of 78.35%; and 2) the local synteny is highly conserved, consisting of the upstream and downstream genes being orthologous to *D. melanogaster* ( Figure 1A ) *.*

***Protein Model***

*Sik3* in *D. mojavensis* has six protein coding isoforms (Sik3-PA, Sik3-PB, Sik3-PC, Sik3, PD, Sik3-PE, Sik3-PF) ( Figure 1B ). mRNA isoform *Sik3-RA* contains seven CDSs. mRNA isoforms *Sik3-RC* , *Sik3-RD* , *Sik3-RB* all contain eleven CDSs, and mRNA isoforms *Sik3-RF* and *Sik3-RE* contain ten CDSs. These isoforms are the same relative to the ortholog in *D. melanogaster* which contains six protein coding isoforms (Sik3-PA, Sik3-PB, Sik3-PC, Sik3, PD, Sik3-PE, Sik3-PF) with the same CDS structure. The dot plot that compares the protein alignment between *D. mojavensis* and *D. melanogaster* shows an indel within the 10th CDS, and the sequence alignment has a percent identity of 78.35% as determined by *blastp* ( Figure 1C ). The coordinates of the curated gene models (Sik3-PC, Sik3-PB, Sik3-PD, Sik3-PE, Sik3-PF and Sik3-PA) can be found in NCBI at GenBank using the accessions BK064485 , BK064486 , BK064487 , BK064488 , BK064489 and BK064490 . These data are also available in Extended Data files below, which are archived in CaltechData.

## Methods

Detailed methods including algorithms, database versions, and citations for the complete annotation process can be found in Rele et al. (2023). Briefly, students use the GEP instance of the UCSC Genome Browser v.435 ( https://gander.wustl.edu ; Kent WJ et al., 2002; Navarro Gonzalez et al., 2021) to examine the genomic neighborhood of their reference IIS gene in the *D. melanogaster* genome assembly (Aug. 2014; BDGP Release 6 + ISO1 MT/dm6). Students then retrieve the protein sequence for the *D. melanogaster* reference gene for a given isoform and run it using *tblastn* against their target *Drosophila* species genome assembly on the NCBI BLAST server ( https://blast.ncbi.nlm.nih.gov/Blast.cgi ; Altschul et al., 1990) to identify potential orthologs. To validate the potential ortholog, students compare the local genomic neighborhood of their potential ortholog with the genomic neighborhood of their reference gene in *D. melanogaster* . This local synteny analysis includes at minimum the two upstream and downstream genes relative to their putative ortholog. They also explore other sets of genomic evidence using multiple alignment tracks in the Genome Browser, including BLAT alignments of RefSeq Genes, Spaln alignment of *D. melanogaster* proteins, multiple gene prediction tracks (e.g., GeMoMa, Geneid, Augustus), and modENCODE RNA-Seq from the target species. Detailed explanation of how these lines of genomic evidenced are leveraged by students in gene model development are described in Rele et al. (2023). Genomic structure information (e.g., CDSs, intron-exon number and boundaries, number of isoforms) for the *D. melanogaster* reference gene is retrieved through the Gene Record Finder ( https://gander.wustl.edu/~wilson/dmelgenerecord/index.html ; Rele et al *.,* 2023). Approximate splice sites within the target gene are determined using *tblastn* using the CDSs from the *D. melanogaste* r reference gene. Coordinates of CDSs are then refined by examining aligned modENCODE RNA-Seq data, and by applying paradigms of molecular biology such as identifying canonical splice site sequences and ensuring the maintenance of an open reading frame across hypothesized splice sites. Students then confirm the biological validity of their target gene model using the Gene Model Checker ( https://gander.wustl.edu/~wilson/dmelgenerecord/index.html ; Rele et al., 2023), which compares the structure and translated sequence from their hypothesized target gene model against the *D. melanogaster* reference gene model. At least two independent models for a gene are generated by students under mentorship of their faculty course instructors. Those models are then reconciled by a third independent researcher mentored by the project leaders to produce the final model. Note: comparison of 5' and 3' UTR sequence information is not included in this GEP CURE protocol.

## Data Availability

Description: A GFF, FASTA, and PEP of the model. Resource Type: Model. DOI: https://doi.org/10.22002/ezqb1-e9755

## References

[R1] Altschul SF, Gish W, Miller W, Myers EW, Lipman DJ (1990). Basic local alignment search tool.. J Mol Biol.

[R2] Choi S, Lim DS, Chung J (2015). Feeding and Fasting Signals Converge on the LKB1-SIK3 Pathway to Regulate Lipid Metabolism in Drosophila.. PLoS Genet.

[R3] Congleton H, Kiser CA, Colom Diaz PA, Schlichting E, Walton DA, Long LJ, Reed LK, Martinez-Cruzado JC, Rele CP (2022). Drosophila mojavensis - chico.. MicroPubl Biol.

[R4] Clark AG, Eisen MB, Smith DR, Bergman CM, Oliver B, Markow TA, Kaufman TC, Kellis M, Gelbart W, Iyer VN, Pollard DA, Sackton TB, Larracuente AM, Singh ND, Abad JP, Abt DN, Adryan B, Aguade M, Akashi H, Anderson WW, Aquadro CF, Ardell DH, Arguello R, Artieri CG, Barbash DA, Barker D, Barsanti P, Batterham P, Batzoglou S, Begun D, Bhutkar A, Blanco E, Bosak SA, Bradley RK, Brand AD, Brent MR, Brooks AN, Brown RH, Butlin RK, Caggese C, Calvi BR, Bernardo de Carvalho A, Caspi A, Castrezana S, Celniker SE, Chang JL, Chapple C, Chatterji S, Chinwalla A, Civetta A, Clifton SW, Comeron JM, Costello JC, Coyne JA, Daub J, David RG, Delcher AL, Delehaunty K, Do CB, Ebling H, Edwards K, Eickbush T, Evans JD, Filipski A, Findeiss S, Freyhult E, Fulton L, Fulton R, Garcia AC, Gardiner A, Garfield DA, Garvin BE, Gibson G, Gilbert D, Gnerre S, Godfrey J, Good R, Gotea V, Gravely B, Greenberg AJ, Griffiths-Jones S, Gross S, Guigo R, Gustafson EA, Haerty W, Hahn MW, Halligan DL, Halpern AL, Halter GM, Han MV, Heger A, Hillier L, Hinrichs AS, Holmes I, Hoskins RA, Hubisz MJ, Hultmark D, Huntley MA, Jaffe DB, Jagadeeshan S, Jeck WR, Johnson J, Jones CD, Jordan WC, Karpen GH, Kataoka E, Keightley PD, Kheradpour P, Kirkness EF, Koerich LB, Kristiansen K, Kudrna D, Kulathinal RJ, Kumar S, Kwok R, Lander E, Langley CH, Lapoint R, Lazzaro BP, Lee SJ, Levesque L, Li R, Lin CF, Lin MF, Lindblad-Toh K, Llopart A, Long M, Low L, Lozovsky E, Lu J, Luo M, Machado CA, Makalowski W, Marzo M, Matsuda M, Matzkin L, McAllister B, McBride CS, McKernan B, McKernan K, Mendez-Lago M, Minx P, Mollenhauer MU, Montooth K, Mount SM, Mu X, Myers E, Negre B, Newfeld S, Nielsen R, Noor MA, O'Grady P, Pachter L, Papaceit M, Parisi MJ, Parisi M, Parts L, Pedersen JS, Pesole G, Phillippy AM, Ponting CP, Pop M, Porcelli D, Powell JR, Prohaska S, Pruitt K, Puig M, Quesneville H, Ram KR, Rand D, Rasmussen MD, Reed LK, Reenan R, Reily A, Remington KA, Rieger TT, Ritchie MG, Robin C, Rogers YH, Rohde C, Rozas J, Rubenfield MJ, Ruiz A, Russo S, Salzberg SL, Sanchez-Gracia A, Saranga DJ, Sato H, Schaeffer SW, Schatz MC, Schlenke T, Schwartz R, Segarra C, Singh RS, Sirot L, Sirota M, Sisneros NB, Smith CD, Smith TF, Spieth J, Stage DE, Stark A, Stephan W, Strausberg RL, Strempel S, Sturgill D, Sutton G, Sutton GG, Tao W, Teichmann S, Tobari YN, Tomimura Y, Tsolas JM, Valente VL, Venter E, Venter JC, Vicario S, Vieira FG, Vilella AJ, Villasante A, Walenz B, Wang J, Wasserman M, Watts T, Wilson D, Wilson RK, Wing RA, Wolfner MF, Wong A, Wong GK, Wu CI, Wu G, Yamamoto D, Yang HP, Yang SP, Yorke JA, Yoshida K, Zdobnov E, Zhang P, Zhang Y, Zimin AV, Baldwin J, Abdouelleil A, Abdulkadir J, Abebe A, Abera B, Abreu J, Acer SC, Aftuck L, Alexander A, An P, Anderson E, Anderson S, Arachi H, Azer M, Bachantsang P, Barry A, Bayul T, Berlin A, Bessette D, Bloom T, Blye J, Boguslavskiy L, Bonnet C, Boukhgalter B, Bourzgui I, Brown A, Cahill P, Channer S, Cheshatsang Y, Chuda L, Citroen M, Collymore A, Cooke P, Costello M, D'Aco K, Daza R, De Haan G, DeGray S, DeMaso C, Dhargay N, Dooley K, Dooley E, Doricent M, Dorje P, Dorjee K, Dupes A, Elong R, Falk J, Farina A, Faro S, Ferguson D, Fisher S, Foley CD, Franke A, Friedrich D, Gadbois L, Gearin G, Gearin CR, Giannoukos G, Goode T, Graham J, Grandbois E, Grewal S, Gyaltsen K, Hafez N, Hagos B, Hall J, Henson C, Hollinger A, Honan T, Huard MD, Hughes L, Hurhula B, Husby ME, Kamat A, Kanga B, Kashin S, Khazanovich D, Kisner P, Lance K, Lara M, Lee W, Lennon N, Letendre F, LeVine R, Lipovsky A, Liu X, Liu J, Liu S, Lokyitsang T, Lokyitsang Y, Lubonja R, Lui A, MacDonald P, Magnisalis V, Maru K, Matthews C, McCusker W, McDonough S, Mehta T, Meldrim J, Meneus L, Mihai O, Mihalev A, Mihova T, Mittelman R, Mlenga V, Montmayeur A, Mulrain L, Navidi A, Naylor J, Negash T, Nguyen T, Nguyen N, Nicol R, Norbu C, Norbu N, Novod N, O'Neill B, Osman S, Markiewicz E, Oyono OL, Patti C, Phunkhang P, Pierre F, Priest M, Raghuraman S, Rege F, Reyes R, Rise C, Rogov P, Ross K, Ryan E, Settipalli S, Shea T, Sherpa N, Shi L, Shih D, Sparrow T, Spaulding J, Stalker J, Stange-Thomann N, Stavropoulos S, Stone C, Strader C, Tesfaye S, Thomson T, Thoulutsang Y, Thoulutsang D, Topham K, Topping I, Tsamla T, Vassiliev H, Vo A, Wangchuk T, Wangdi T, Weiand M, Wilkinson J, Wilson A, Yadav S, Young G, Yu Q, Zembek L, Zhong D, Zimmer A, Zwirko Z, Jaffe DB, Alvarez P, Brockman W, Butler J, Chin C, Gnerre S, Grabherr M, Kleber M, Mauceli E, MacCallum I, Drosophila 12 Genomes Consortium. (2007). Evolution of genes and genomes on the Drosophila phylogeny.. Nature.

[R5] Durando CM, Baker RH, Etges WJ, Heed WB, Wasserman M, DeSalle R (2000). Phylogenetic analysis of the repleta species group of the genus Drosophila using multiple sources of characters.. Mol Phylogenet Evol.

[R6] Funato H, Miyoshi C, Fujiyama T, Kanda T, Sato M, Wang Z, Ma J, Nakane S, Tomita J, Ikkyu A, Kakizaki M, Hotta-Hirashima N, Kanno S, Komiya H, Asano F, Honda T, Kim SJ, Harano K, Muramoto H, Yonezawa T, Mizuno S, Miyazaki S, Connor L, Kumar V, Miura I, Suzuki T, Watanabe A, Abe M, Sugiyama F, Takahashi S, Sakimura K, Hayashi Y, Liu Q, Kume K, Wakana S, Takahashi JS, Yanagisawa M (2016). Forward-genetics analysis of sleep in randomly mutagenized mice.. Nature.

[R7] Gramates L Sian, Agapite Julie, Attrill Helen, Calvi Brian R, Crosby Madeline A, dos Santos Gilberto, Goodman Joshua L, Goutte-Gattat Damien, Jenkins Victoria K, Kaufman Thomas, Larkin Aoife, Matthews Beverley B, Millburn Gillian, Strelets Victor B, Perrimon Norbert, Gelbart Susan Russo, Agapite Julie, Broll Kris, Crosby Lynn, dos Santos Gil, Falls Kathleen, Gramates L Sian, Jenkins Victoria, Longden Ian, Matthews Beverley, Seme Jolene, Tabone Christopher J, Zhou Pinglei, Zytkovicz Mark, Brown Nick, Antonazzo Giulia, Attrill Helen, Garapati Phani, Goutte-Gattat Damien, Larkin Aoife, Marygold Steven, McLachlan Alex, Millburn Gillian, Öztürk-Çolak Arzu, Pilgrim Clare, Trovisco Vitor, Calvi Brian, Kaufman Thomas, Goodman Josh, Krishna Pravija, Strelets Victor, Thurmond Jim, Cripps Richard, Lovato TyAnna, the FlyBase Consortium (2022). FlyBase: a guided tour of highlighted features. Genetics.

[R8] Grewal SS (2008). Insulin/TOR signaling in growth and homeostasis: a view from the fly world.. Int J Biochem Cell Biol.

[R9] Hietakangas V, Cohen SM (2009). Regulation of tissue growth through nutrient sensing.. Annu Rev Genet.

[R10] Jenkins VK, Larkin A, Thurmond J, FlyBase Consortium (2022). Using FlyBase: A Database of Drosophila Genes and Genetics.. Methods Mol Biol.

[R11] Kent WJ, Sugnet CW, Furey TS, Roskin KM, Pringle TH, Zahler AM, Haussler D (2002). The human genome browser at UCSC.. Genome Res.

[R12] Larkin A, Marygold SJ, Antonazzo G, Attrill H, Dos Santos G, Garapati PV, Goodman JL, Gramates LS, Millburn G, Strelets VB, Tabone CJ, Thurmond J, FlyBase Consortium. (2021). FlyBase: updates to the Drosophila melanogaster knowledge base.. Nucleic Acids Res.

[R13] Liu Z, Jiang L, Li C, Li C, Yang J, Yu J, Mao R, Rao Y (2022). LKB1 is physiologically required for sleep from Drosophila melanogaster to the Mus musculus.. Genetics.

[R14] Mudge JM, Harrow J (2016). The state of play in higher eukaryote gene annotation.. Nat Rev Genet.

[R15] Myers A, Hoffman A, Natysin M, Arsham AM, Stamm J, Thompson JS, Rele CP, Reed LK (2024). Gene model for the ortholog Myc in Drosophila ananassae.. MicroPubl Biol.

[R16] Navarro Gonzalez J, Zweig AS, Speir ML, Schmelter D, Rosenbloom KR, Raney BJ, Powell CC, Nassar LR, Maulding ND, Lee CM, Lee BT, Hinrichs AS, Fyfe AC, Fernandes JD, Diekhans M, Clawson H, Casper J, Benet-Pagès A, Barber GP, Haussler D, Kuhn RM, Haeussler M, Kent WJ (2021). The UCSC Genome Browser database: 2021 update.. Nucleic Acids Res.

[R17] Okamoto M, Takemori H, Katoh Y (2004). Salt-inducible kinase in steroidogenesis and adipogenesis.. Trends Endocrinol Metab.

[R18] Patterson JT and JF Crow, 1940. Hybridization in the mulleri group of Drosophila. *Univ. Texas Publs* , 4032, 167-189.

[R19] Raney BJ, Dreszer TR, Barber GP, Clawson H, Fujita PA, Wang T, Nguyen N, Paten B, Zweig AS, Karolchik D, Kent WJ (2013). Track data hubs enable visualization of user-defined genome-wide annotations on the UCSC Genome Browser.. Bioinformatics.

[R20] Rele Chinmay P., Sandlin Katie M., Leung Wilson, Reed Laura K. (2023). Manual annotation of Drosophila genes: a Genomics Education Partnership protocol. F1000Research.

[R21] Tello-Ruiz MK, Marco CF, Hsu FM, Khangura RS, Qiao P, Sapkota S, Stitzer MC, Wasikowski R, Wu H, Zhan J, Chougule K, Barone LC, Ghiban C, Muna D, Olson AC, Wang L, Ware D, Micklos DA (2019). Double triage to identify poorly annotated genes in maize: The missing link in community curation.. PLoS One.

[R22] Walkinshaw DR, Weist R, Kim GW, You L, Xiao L, Nie J, Li CS, Zhao S, Xu M, Yang XJ (2013). The tumor suppressor kinase LKB1 activates the downstream kinases SIK2 and SIK3 to stimulate nuclear export of class IIa histone deacetylases.. J Biol Chem.

[R23] Wang B, Moya N, Niessen S, Hoover H, Mihaylova MM, Shaw RJ, Yates JR 3rd, Fischer WH, Thomas JB, Montminy M (2011). A hormone-dependent module regulating energy balance.. Cell.

[R24] Wasserman, M. (1992). Cytological evolution of the Drosophila repleta species group. *Krimbas, Powell, 1992* : 455-552. FBrf0063954

[R25] Wehr MC, Holder MV, Gailite I, Saunders RE, Maile TM, Ciirdaeva E, Instrell R, Jiang M, Howell M, Rossner MJ, Tapon N (2013). Salt-inducible kinases regulate growth through the Hippo signalling pathway in Drosophila.. Nat Cell Biol.

